# Contemporary global practice in dissection and closure techniques for lower abdominal transverse incisions: a cross-sectional survey

**DOI:** 10.3389/jaws.2026.17006

**Published:** 2026-09-04

**Authors:** Leo R. Brown, Hephzibah Adegoke, Ellen Gardner, Thomas M. Drake, James Lucocq, Artur Zanellato

**Affiliations:** 1 Department of Clinical Surgery, University of Edinburgh, Royal Infirmary of Edinburgh, Edinburgh, United Kingdom; 2 Edinburgh Medical School, University of Edinburgh, Royal Infirmary of Edinburgh, Edinburgh, United Kingdom; 3 Department of General Surgery, Dumfries and Galloway Royal Infirmary, Dumfries, United Kingdom; 4 Centre for Medical Informatics, Usher Institute, University of Edinburgh, Edinburgh, United Kingdom

**Keywords:** caesarean section, hernia, LATI, LATIH, surgical technique

## Abstract

**Purpose:**

To characterise international contemporary practice in dissection and closure of lower abdominal transverse incisions (LATI) across surgical specialties.

**Methods:**

An open, cross-sectional survey of surgeons with experience of performing LATI independently was conducted using an anonymised online questionnaire disseminated via professional networks. Data were captured pertaining to respondent characteristics, operative techniques for dissection and closure of abdominal layers, and experience with incisional hernias related to these incisions.

**Results:**

Overall, 231 respondents from 31 countries across 6 continents were included, following relevant exclusions. Respondents comprised general surgeons (68.4%), obstetricians and gynaecologists (O&G; 26.4%) and urologists (5.2%). O&G respondents performed a significantly higher annual LATI case volume, when compared to other specialists (>50 cases/year: 60.7% vs. 6.5%, *p* < 0.001). Peritoneal entry varied between O&G and other surgeons, with O&G more commonly using blunt peritoneal entry (62.3% vs. 7.6%, *p* < 0.001) and a transverse, rather than longitudinal, peritoneal opening (54.1% vs. 15.9%, *p* < 0.001). Rates of peritoneal non-closure were higher amongst O&G compared to other subspecialties (77.0% vs. 24.1%, *p* < 0.001). More frequent use of a ‘small bites’ fascial closure technique was noted among general surgeons and urologists (85.3%), when compared to O&G (42.6%, *p* < 0.001). Most respondents considered LATI hernias to be underreported (84.0%), however more general surgeons had encountered LATI hernias (general surgery: 85.4%, urology: 41.7% and O&G: 42.6%, *p* < 0.001).

**Conclusion:**

There is substantial inter-specialty variation in closure techniques for LATI. Differences in practice may influence incisional hernia development and reporting, highlighting a need for further outcome-focused research and standardisation.

## Introduction

The type of incision employed in abdomino-pelvic surgery is largely dependent on the required operative access, alongside the surgeon’s experience and preferences [1]. The perceived risk of postoperative morbidity may also influence choice, with different incision types and closure methods known to be associated with varying risks of complications [2]. These may include incisional hernias, wound dehiscence, and postoperative pain, all of which can contribute substantially to patient morbidity and subsequent quality of life. Incision and closure techniques remain relatively heterogeneous across most abdomino-pelvic surgeries, with the exception of caesarean sections (c-section) where the use of a lower abdominal transverse incision (LATI) is now almost universal [3]. The preference for the LATI approach over a midline incision may reflect a more favourable cosmetic appearance and a lower risk of incisional hernias [1, 4]. Another notable advantage is decreased postoperative pain, which is in turn associated with the development of chronic pain. This is particularly important given the increasing rates of c-sections worldwide [5], as younger women with dependent children would bear a disproportionate burden.

There has been an evolution of the LATI approach over time, with notable variations in both incision and closure techniques [6, 7]. While extensive research has supported the use of LATIs, there remains an inconsistent use of terminology in existing literature regarding exactly what entry and closure approaches have been utilised. Previous studies have explored closure techniques of midline abdominal incisions and their uptake among surgeons [8], however, there remains a paucity of research specifically evaluating LATI practices. Methods for abdominal access and closure represent modifiable factors that may influence rates of postoperative complications. Of particular concern are interparietal hernias, which may remain clinically occult within the abdominal wall layers, and whose relationship with LATI entry and closure techniques remains poorly understood. As such, it is important to ascertain how practices may differ between the various surgical specialties that use LATI, or across the world.

This structured survey aims to characterise contemporary international practice for access and closure of LATI and explore variations that may be evident between surgical subspecialties or geographic regions.

## Materials and methods

This study was a cross-sectional, questionnaire-based online electronic survey pertaining to surgical techniques used for access and closure via transverse incisions across the lower abdomen. Participation was entirely voluntary with informed consent implied by completion of the survey. The work did not involve patients, patient data, or any clinical intervention, no identifiable personal data were collected. All results were anonymised. In line with UK Health Research Authority guidance, formal Research Ethics Committee approval was therefore not required. Findings were reported in accordance with the Checklist for Reporting Results of Internet E-Surveys (CHERRIES) guidance [9].

### Study population

The target population included all surgeons with experience of independently performing intra-abdominal surgical procedures through a LATI or managing resultant hernias from these incisions. Clinicians from all surgical subspecialties were eligible to participate, including upper and lower gastrointestinal surgery, hepatobiliary and pancreatic surgery, other general subspecialists, obstetrics and gynaecology and urology. Respondents were categorised by subspecialty, based on broad surgical training pathways (general surgery vs. urology vs. obstetrics and gynaecology). Relationships between LATI technique and respondents’ country of clinical practice were also explored, with countries grouped based on income status. This was defined according to World Bank Analytical Classification based on gross national income *per capita* [10]. Countries were categorised as either high-income country (HIC), upper-middle income country (UMIC), lower-middle income country (LMIC) or low-income country (LIC).

### Questionnaire development, content and pre-testing

The questionnaire was developed by the study investigators based on review of the relevant existing literature, and consultation with senior surgeons from relevant specialties. Pilot testing was conducted across a small group of surgeons prior to dissemination, with feedback used to refine the questionnaire. Adaptive questioning was used, based on responses to early questions, to reduce the time taken for participants to complete it. Mandatory response functions were used for other questions to minimise missing data. Pilot responses were not included in the final analysis. Questions pertained to respondent characteristics, surgical practice for dissection and closure of abdominal layers with LATI, and experience with hernias related to these incisions. The resultant questionnaire distributed can be found in Supplementary Appendix 1.

### Survey administration

Data were collected using a secure, electronic questionnaire hosted on ‘Google Forms’. Advertisement and invitations to participate were disseminated nationally and internationally via professional surgical mailing lists, social media platforms and collaborator networks. Recipients were also encouraged to share the survey within their own professional networks. Responses were collected across a 10-month period (January to October 2025). Email addresses were collected to check for duplicate responses, which were deleted prior to data transfer and analysis.

### Data handling and statistical analysis

Survey responses were collected in an anonymised fashion and data were exported to a secure server hosted at University of Edinburgh for analysis. Descriptive statistics were used to summarise responses with categorical variables reported as frequencies and percentages. Continuous data were summarized as mean (standard deviation) or median (inter-quartile range) based on visual and statistical evaluation for normality. Subsequent comparisons between groups were conducted using appropriate parametric or non-parametric tests. Categorical data were cross-tabulated, and differences tested using χ ^2^ or Fisher’s exact test. R 4.2.2 (R Foundation for Statistical Computing, Vienna, Austria) was used for all data visualisation and analyses with *dplyr, ggplot2, finalfit, forcats, rnaturalearth* and *sf* packages.

## Results

Overall, 240 responses were collected across the 10-month data collection period. Amongst these, 6 duplicate responses were identified. A further 3 were ineligible as those respondents indicated that they did not have experience of independently performing intra-abdominal surgical procedures through a LATI. This resulted in a final sample of 231 responses, used for all subsequent analyses.

**FIGURE 1:**
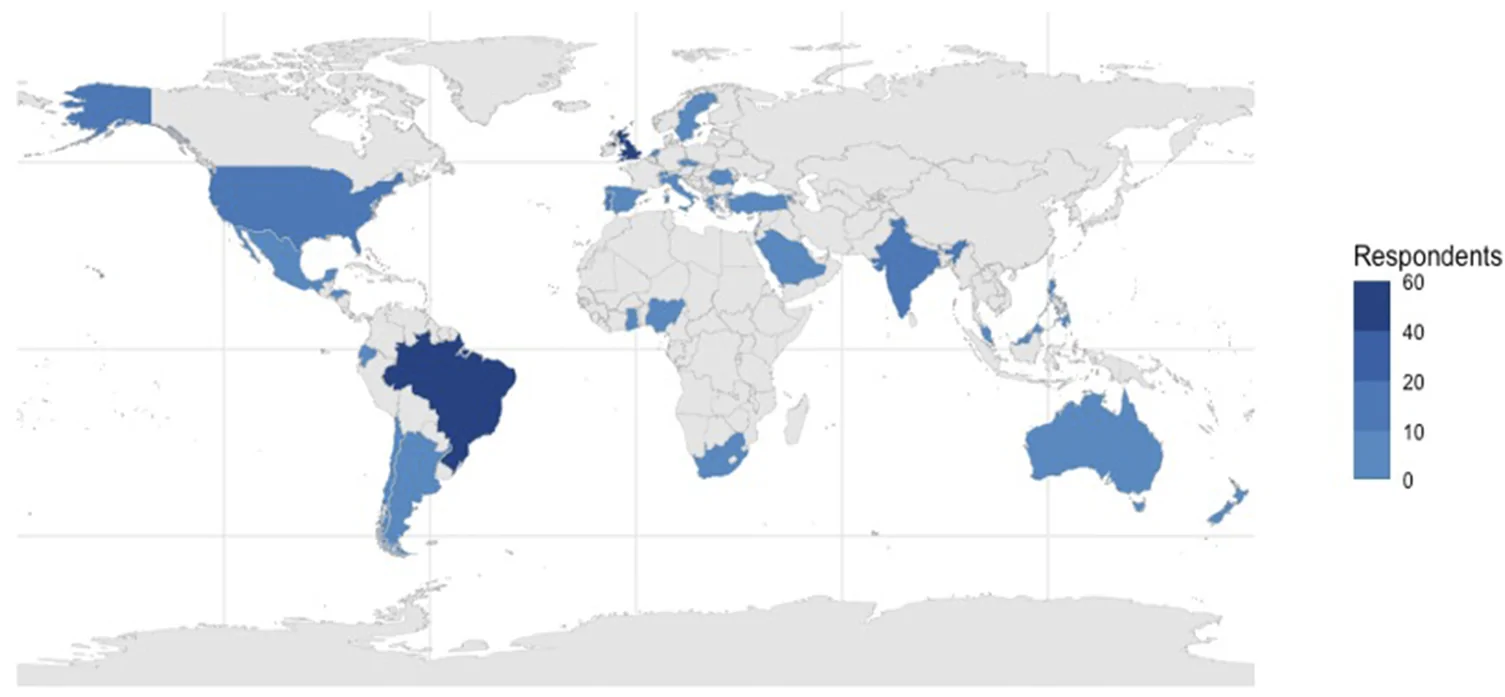
Global distribution of survey respondents. Respondent Countries: Argentina, Armenia, Australia, Brazil, Chile, Czech Republic, Ecuador, France, Ghana, Greece, Honduras, India, Israel, Italy, Malaysia, Mexico, Netherlands, New Zealand, Nigeria, Norway, Philippines, Portugal, Saudi Arabia, South Africa, Spain, Sweden, Switzerland, Turkey, United Kingdom, United States of America.

The 231 respondents included clinicians practicing in 31 countries across 6 continents (Figure 1). There was a median age of 46 years (IQR: 39–55) and 68.8% were male. Subspecialty interests varied across the sample with a preponderance of general surgeons (hernia surgery: n = 86, upper gastrointestinal surgery: n = 27, colorectal surgery: n = 19, emergency or other general surgery subspecialist: n = 16), with obstetricians and gynaecologists (O&G) (n = 61) and urologists (n = 12) also well represented. These were dichotomised (Table 1; general or urological surgeons [73.6%] and OG [26.4%]), and trichotomised (Supplementary Appendix 2; general surgeons [68.4%], O&G [26.4%] and urologists [5.2%]). Respondents were also grouped by country income status (Supplementary Appendix 3; HIC [50.2%], UMIC [39.0%] and LMIC [10.8%]). Respondents had a median of 15 years consultant experience (IQR: 5–23). The annual number of LATI cases performed varied across the study cohort (0–10 cases; n = 115 [49.8%], 10–20 cases; n = 32 [13.9%], 20–50 cases; n = 36 [15.6%] and >50 cases; n = 48 [20.8%]).

**TABLE 1:** Demographics and experience compared by respondent subspecialty.

Variable	​	General surgery & urology (n = 170)	Obstetrics & gynaecology (n = 61)	*p* value
Age (years)	Median (IQR)	47.0 (40.0–55.0)	44.0 (37.5–53.5)	0.153
Gender	Male	138 (81.2)	21 (34.4)	<0.001
​	Female	32 (18.8)	40 (65.6)	​
Country income	HIC	76 (44.7)	40 (65.6)	0.015
	UMIC	72 (42.4)	18 (29.5)	​
	LMIC	22 (12.9)	3 (4.9)	​
Consultant experience (years)	Median (IQR)	16.0 (7.0–23.0)	9.0 (2.0–18.0)	0.004
LATI case frequency (cases/year)	0–10	109 (64.1)	6 (9.8)	<0.001
	10–20	27 (15.9)	5 (8.2)	​
	20–50	23 (13.5)	13 (21.3)	​
	>50	11 (6.5)	37 (60.7)	​

Data are displayed as number (%) unless stated otherwise. HIC: high income country, UMIC: Upper-Middle Income Country, LMIC: Lower-Middle Income Country. LATI: Lower Abdominal Transverse Incision. experience reported as years in independent consultant practice.

### Respondent demographics

Age was comparable across subspecialty respondent groups, but a male preponderance was noted in general and urological surgeons (81.2%), while most O&G respondents were female (65.6%, *p* < 0.001). A longer duration of consultant practice was evident in the general and urological surgeons (median: 16 years), when compared to O&G (median: 9 years, *p* = 0.004). There was a higher annual case volume using a LATI incision amongst obstetrics and gynaecology (>50 cases/year: 60.7%) when compared to all other subspecialties (>50 cases/year: 6.5%, *p* < 0.001).

### LATI entry technique

Diathermy was the preferred method of entry into the fascia among general surgeons and urologists (58.2%), while most O&G specialists favoured a combination of dissection modalities (O&G: 47.5%, *p* < 0.001, Table 2). All groups predominantly separated the rectus muscles after detaching from the anterior sheath, but different techniques were used to open the peritoneum. There were higher rates of blunt dissection among O&G compared to other subspecialties (62.3% vs. 7.6%, *p* < 0.001), where sharp dissection was the predominant method among general surgeons (44.1%, *p* < 0.001). The direction of peritoneum opening also differed between subspecialties, with transverse opening more frequently used in O&G (54.1%) where general surgery and urology most frequently opt for a longitudinal opening (84.1%, *p* < 0.001).

**TABLE 2:** LATI entry technique.

Variable	​	General surgery & urology (n = 170)	Obstetrics & gynaecology (n = 61)	*p* value
Fascia	Blunt	3 (1.8)	10 (16.4)	<0.001
Opening	Sharp	25 (14.7)	14 (23.0)	​
​	Diathermy	99 (58.2)	8 (13.1)	​
​	Combination	43 (25.3)	29 (47.5)	​
Rectus	Partly cut	9 (5.3)	1 (1.6)	0.019
Opening	Entirely cut	1 (0.6)	3 (4.9)	​
​	Separate & detached	92 (54.1)	41 (67.2)	​
​	Separate & attached	68 (40.0)	16 (26.2)	​
Peritoneum	Blunt	13 (7.6)	38 (62.3)	<0.001
Opening	Sharp	75 (44.1)	12 (19.7)	​
​	Diathermy	48 (28.2)	2 (3.3)	​
​	Combination	34 (20.0)	9 (14.8)	​
Peritoneum	Transverse	27 (15.9)	33 (54.1)	<0.001
Direction	Longitudinal	143 (84.1)	28 (45.9)	​

Data are displayed as number (%) unless stated otherwise. LATI: lower abdominal transverse incision.

### LATI closure technique

Significantly higher rates of peritoneal non-closure were noted amongst O&G compared to other subspecialties (77.0% vs. 24.1%, *p* < 0.001, Table 3). Similarly, O&G clinicians were less likely to re-approximate the rectus muscle (O&G: 36.1% vs. others: 58.2%, *p* = 0.005). Urologists had the highest rates of re-approximating the rectus muscle across the subspecialty groups (Supplementary Appendix 6; 83.3%, *p* < 0.002). Clinicians practicing in UMIC and LMIC had higher rates of re-approximating the rectus muscle in comparison to their counterparts in HIC (Supplementary Appendix 7; UMIC: 75.6% vs. LMIC: 56% vs. HIC: 33.6, *p* < 0.001). Technique for fascial closure also differed between groups, with more frequent use of a ‘small bites’ technique amongst general surgeons and urologists (general surgery and urology: 85.3% vs. 42.6%, *p* < 0.001). Continuous, rather than interrupted closure, was predominant for all respondent groups. No association was evidence for suture material to close the fascia, with slowly absorbable sutures being the preferred option throughout (general surgery and urology: 82.9%, O&G: 80.3%, *p* < 0.001).

**TABLE 3:** LATI closure technique.

Variable	​	General surgery & urology (n = 170)	Obstetrics & gynaecology (n = 61)	*p* value
Peritoneum	Yes	129 (75.9)	14 (23.0)	<0.001
Close	No	41 (24.1)	47 (77.0)	​
Suture	Fast absorbable	62 (36.5)	15 (24.6)	0.098
Peritoneum	Slow absorbable	71 (41.8)	6 (9.8)	​
​	Non-absorbable	1 (0.6)	0 (0.0)	​
​	(Missing)	36 (21.2)	40 (65.6)	​
Close rectus	Yes	99 (58.2)	22 (36.1)	0.005
​	No	71 (41.8)	39 (63.9)	​
Suture rectus	Fast absorbable	45 (26.5)	15 (24.6)	0.194
​	Slow absorbable	60 (35.3)	9 (14.8)	​
​	Non-absorbable	1 (0.6)	0 (0.0)	​
​	(Missing)	64 (37.6)	37 (60.7)	​
Close fascia	Continuous	165 (97.1)	57 (93.4)	0.386
​	Interrupted	5 (2.9)	4 (6.6)	​
Fascia method	Small bites	145 (85.3)	26 (42.6)	<0.001
​	Large bites	21 (12.4)	32 (52.5)	​
​	(Missing)	4 (2.4)	3 (4.9)	​
Fascia suture	Fast absorbable	2 (1.2)	11 (18.0)	<0.001
​	Slow absorbable	141 (82.9)	49 (80.3)	​
​	Non-absorbable	27 (15.9)	1 (1.6)	​

Data are displayed as number (%) unless stated otherwise. LATI: lower abdominal transverse incision.

### Experience of LATI hernias

The majority of general surgeons have encountered a LATI hernia (82.4%), where these have less commonly observed by urology (41.7%) and O&G subspecialists (42.6%, *p* < 0.001, Table 4). Most respondents agreed that these are underreported (84.0%), however the proportion who held this opinion was significantly higher in general surgeons (*p* = 0.018). Across all subspecialties, most surgeons encounter between 0 and 5 LATI hernias each year (75%), however a significantly higher rate of identification was reported by general surgeons (*p* < 0.001).

**TABLE 4:** LATI hernias.

Variable	​	General surgery (n = 158)	Urology surgery (n = 12)	Obstetrics & gynaecology (n = 61)	p value
LATI hernia	Yes	135 (85.4)	5 (41.7)	26 (42.6)	<0.001
Seen previously	No	23 (14.6)	7 (58.3)	35 (57.4)	​
Number of LATI	0–5	109 (69.0)	10 (83.3)	55 (90.2)	<0.001
Hernias seen	5–10	30 (19.0)	1 (8.3)	1 (1.6)	​
(per year)	>10	19 (12.0)	0 (0.0)	0 (0.0)	​
​	(Missing)	0 (0.0)	1 (8.3)	5 (8.2)	​
LATI hernia	Total	31 (19.6)	0 (0.0)	4 (6.6)	<0.001
Type seen	Partial	58 (36.7)	9 (75.0)	34 (55.7)	​
​	Mixed	69 (43.7)	2 (16.7)	13 (21.3)	​
​	(Missing)	0 (0.0)	1 (8.3)	10 (16.4)	​
Are LATI hernias	Yes	144 (91.1)	8 (66.7)	42 (68.9)	0.018
Underreported	No	14 (8.9)	4 (33.3)	9 (14.8)	​
​	(Missing)	0 (0.0)	0 (0.0)	10 (16.4)	​
LATI hernia found	Commonly not reported	68 (43.0)	5 (41.7)	32 (52.5)	0.048
(CT imaging)	Commonly reported (report not as visualised)	31 (19.6)	1 (8.3)	3 (4.9)	​
​	Commonly reported (report as visualised)	59 (37.3)	5 (41.7)	14 (23.0)	​
​	(Missing)	0 (0.0)	1 (8.3)	12 (19.7)	​
LATI hernia found	Yes	98 (62.0)	4 (33.3)	18 (29.5)	<0.001
(Laparoscopy)	No	60 (38.0)	8 (66.7)	38 (62.3)	​
​	(Missing)	0 (0.0)	0 (0.0)	5 (8.2)	​

Data are displayed as number (%) unless stated otherwise. LATI: Lower Abdominal Transverse Incision. CT: computed tomography.

The type of LATI hernias encountered differed across specialties. While general surgeons commonly encountered a mix of total and partial (inter-parietal) hernias (43.7%), urologists and obstetricians and gynaecologists predominantly reported patients with partial hernias (urology: 75%, O&G: 55.7%, *p* < 0.001). Perceived detection and reporting of LATI hernias via CT also differed between the subspecialty groups (*p* = 0.048). Most general surgeons report that they have found LATI hernias incidentally during laparoscopy (62.0%) while urologists (33.3%) and O&G specialists (29.5%), less commonly report this finding (*p* < 0.001).

## Discussion

This cross-sectional survey captured international, pan-specialty data pertaining to entry and closure techniques for LATI. It is the first study to address this question and characterise contemporary variation in practice for this increasingly used incision. Key findings include the differences in rates of small bite fascial closure and non-closure of the peritoneum between O&G and other subspecialty surgeons. Most respondents have encountered incidental LATI hernias, commonly inter-parietal in nature, and concerns were raised that these hernias remain commonly overlooked and underreported. Further investigation into the true prevalence, clinical importance and potential preventability of LATI hernias is merited.

A higher case volume using LATI was noted for O&G clinicians in comparison to general surgeons and urologists. This is an unsurprising finding given that c-sections are among the most commonly performed abdominal surgeries worldwide, with rates continuing to increase [5]. In the United Kingdom (UK) national guidance via NICE recommends the routine use of a LATI incision for c-section [3]. This report noted the numerous eponymous techniques reported for LATI [11–13], and their subsequent variations, and the resultant potential for confusion in distinguishing between these subtly different methods. These recommendations are mirrored internationally by other organisations such as the International Federation of Gynaecology and Obstetrics (FIGO) [14], the European Society of Regional Anaesthesia and Obstetric Anaesthetists Association via the PROSPECT guidelines [15]. However, LATI should also be considered as a valuable option for other surgical subspecialists requiring abdomino-pelvic access, including for specimen extraction. Understanding the current practice of LATIs across various subspecialties and considering entry and closure techniques to gauge commonalities and differences, is therefore highly informative for a wide range of surgeons.

Findings from the present study highlight that general surgeons and urologists tend to open the fascia using diathermy, as opposed to the combination of blunt and sharp dissection used by O&G. Sharp dissection in a longitudinal direction was more commonly used by general surgeons to incise the peritoneum, while blunt dissection in a transverse direction was the predominant peritoneal entry technique employed by O&G specialists. This practice aligns with the NICE guidelines for c-sections, which advise blunt dissection of all tissue layers with selective sharp dissection in cases where previous scar tissue makes blunt dissection too difficult [3]. These guidelines are in keeping with the contemporary modified Joel-Cohen technique, popularised by Stark approximately 30 years ago [13]. The O&G respondents’ preference towards a transverse opening of the parietal peritoneum is similarly in keeping with the Joel-Cohen approach to LATI in an attempt to avoid injury to the bladder [12]. The predominant use of a longitudinal peritoneal opening by general and urological surgeons is more in keeping with the traditional Pfannenstiel incision [11], or indeed abdominal entry via the more commonly utilised midline incision. Inter-specialty differences in practice must be interpreted in context and may be justified by variation in operative exposure requirements, underlying pathology and the potential need for more emergent access in obstetric surgery. Accordingly, rather than representing sub-optimal practice, this variation underscores the importance of understanding rationale for specialty-specific technique across shared anatomical planes. While it is plausible that differences in method of dissection may influence the integrity of muscle layers, and subsequent patterns of hernia formation, the present study cannot establish a causal relationship between entry technique and this outcome.

A key finding of this survey was the variation in practice regarding closure of the parietal peritoneum in LATI. While only a minority of O&G specialists routinely close the peritoneum (23.0%), most general and urological surgeons do (75.9%, *p* < 0.001). Similar findings were observed for the two groups regarding re-approximation of the rectus muscle. This non-closure of the peritoneum aligns with the NICE [3] and PROSPECT [15] recommendations for c-sections. The rationale to these guidelines is that non-closure of parietal peritoneum decreases the operating time and is associated with decreased analgesic requirements. The two largest randomised controlled trials that have addressed this question are CAESAR [16] and CORONIS [17]. Both of these demonstrated equivalence between closure and non-closure across all considered outcomes, including rates of postoperative infection, bleeding and pain scores. However, some potentially important outcomes such as adhesion and hernia formation, remain under-explored. Systematic review of this comparatively sparse evidence base suggests that non-closure may be associated with increased risk of adhesions [18]. While limited and low-quality evidence precludes firm conclusion regarding incisional hernia risk, incomplete restoration of the deeper abdominal wall layers could theoretically permit herniation between tissue planes without producing a conventional, and more clinically evident, full thickness defect. It should also be noted that existing evidence is derived almost exclusively from obstetric populations, and the applicability of trial findings to LATI in other surgical contexts remains uncertain. The rationale for non-closure, as per current guidelines, may need reconsideration were there to be a difference in rates of these outcomes.

Across all the subspecialty groups, the most popular method of fascia closure was continuous slow-absorbable sutures. Differences were evident however, as more general surgeons and urologists opted for a ‘small-bites’ technique (85.3%), while most O&G clinicians employed large bites (52.5%, *p* < 0.001). The European Hernia Society and American Hernia Society are clear in their recommendation for the use of continuous small bites for closure of midline abdominal incisions [4], however again the evidence base for optimal fascial closure in LATI is far more limited, and further investigation is still required.

Several limitations of this study should be acknowledged. The survey was circulated to national and international colleagues across numerous societies and professional networks to enable broad geographical and professional participation, with recipients encouraged to share it further. Although this open, network-based approach increased the survey’s reach, no central distribution log was maintained. As such, neither every dissemination route nor the number and characteristics of individuals who received the invitation but did not participate could be identified and a response rate could not be calculated. Voluntary participation may also have introduced selection bias by favouring individuals connected to these networks or interested in the topic. Consequently, some subspecialties or geographical regions may be over- or under-represented. No weighting or other statistical correction was applied to address potential differences between the study sample and the wider professional population. Further work may be required to establish the external validity of the findings beyond this diverse voluntary-response sample. The survey was designed to be concise, accessible, and relevant to all participating subspecialties, and consequently it did not capture how respondents’ LATI techniques might vary between specific procedures or according to operative urgency. For example, O&G clinicians may use different LATI approaches during elective gynaecological surgery and an emergency caesarean section, which have distinct operative requirements, including in relation to use of diathermy. Combining these contexts may obscure clinically relevant differences and limit the interpretation of the pooled findings. As the available data for the present study do not permit separate analyses, the results should be regarded as hypothesis-generating, with procedure-specific questions requiring investigation through more controlled study designs.

General surgeons were more likely to encounter LATI hernias in comparison to urologists and O&G clinicians, and these hernias tended to be of mixed (total vs. inter-parietal) types. This is perhaps unsurprising as the subspecialty most likely to investigate and manage hernias, would be expected to have a heightened awareness of such pathologies. The vast majority of general surgeons believe LATI hernias are underreported (91.1%). Further work to estimate the true prevalence and clinical relevance of these hernias, including the more subtle inter-parietal hernias, is warranted. Any potential association between LATI entry and closure techniques and hernia formation would similarly be of considerable interest.

## Conclusion

This international, pan-specialty survey demonstrated substantial variation in both entry and closure techniques for LATI. Differences between specialties are particularly evident for peritoneal and fascial closure. While non-closure of the peritoneum aligns with current obstetric evidence and guidelines, the applicability of these guidelines beyond c-section remains uncertain, and key outcomes such as adhesion and hernia formation remain under-explored. Increased awareness and education regarding LATI hernias would be valuable for all surgical subspecialists.

## Data Availability

The raw data supporting the conclusions of this article will be made available by the authors, without undue reservation.
