# Supplementary material for: Contemporary global practice in dissection and closure techniques for lower abdominal transverse incisions: a cross-sectional survey
Source: J Abdom Wall Surg. 2026 Sep 4;5:17006. doi: 10.3389/jaws.2026.17006 (PMC13585698; doi:10.3389/jaws.2026.17006)
Supplement: Supplementary file 1 [file Supplementaryfile1.docx]

**Appendix 1 – Study Questionnaire**

1. In which country do you practice?

_____________________________________ [Short Free Text Response]

2. To which gender do you most identify?

□	Male

□	Female

□	Prefer not to say

□	Other

_____________________________________ [Short Free Text Response]

# 3. How old are you? (years)

_____________________________________ [Short Free Text Response]

4. Which of the following best describes your primary area of surgical practice?

□	Emergency General Surgery

□	General Surgery (Hernia Subspecialist)

□	General Surgery (Upper GI / Bariatric / HPB Subspecialist)

□	General Surgery (Colorectal Subspecialist)

□	General Surgery (Other Subspecialist)

□	Urology

□	Obstetrics & Gynaecology

# 5. How many years have you been practicing as a consultant?

_____________________________________ [Short Free Text Response]

6. Do you independently perform surgical procedures through a lower transverse incision in the abdomen?

□	Yes

□	No

# 7. How often do you use a lower transverse incision in the abdomen?

□	0-10 cases per year

□	10-20 cases per year

□	20-50 cases per year

□	Over 50 cases per year

# Please respond the following question how you would carry out a lower transverse incision in the abdomen if it is not a procedure you typically conduct.

8. How do you routinely open the fascia?

□	Sharp dissection (scalpel or scissors)

□	Diathermy dissection

□	Blunt dissection (finger or instrumental tearing)

□	Combination of sharp, diathermic, and blunt dissection

# 9. How do you routinely handle the rectus muscles?

□	Cut rectus muscles partly

□	Cut rectus sheath entirely

□	Separate rectus muscles after detaching from the anterior rectus sheath

□	Separate rectus muscles without detaching from the anterior rectus sheath

# 10. How do you routinely open the peritoneum?

□	Sharp dissection (scalpel or scissors)

□	Diathermy dissection

□	Blunt dissection (finger or instrumental tearing)

□	Combination of sharp, diathermic, and blunt dissection

# 11. In which direction is the peritoneum opened?

□	Longitudinal

□	Transverse

# 12. Do you routinely close the peritoneum below the arcuate line?

□	Yes

□	No

# 13. If so, what suture material do you use for closure of the peritoneum?

□	Fast absorbable

□	Slowly absorbable

□	Non-absorbable

# 14. Do you routinely re-approximate the rectus muscle using sutures?

□	Yes

□	No

# 15. If so, what suture material do you use to re-approximate the rectus muscle?

□	Fast absorbable

□	Slowly absorbable

□	Non-absorbable

# 16. How do you routinely close the anterior fascia?

□	Continuous suture

□	Interrupted sutures

# 17. Which best describes the suture technique that you routinely use when closing the fascia in a continuous fashion?

□	Small bites suture technique (continuous suture technique where the stitches are placed 5-8mm from the wound edges and 5 mm apart)

□	Large bites suture technique (continuous suture technique where the stitches are placed 10mm or more from the wound edges and 10mm or more apart)

# 18. What suture material do you use for closing the anterior fascia?

□	Fast absorbable

□	Slowly absorbable

□	Non-absorbable

# 19. Have you ever seen a hernia resulting from a lower transverse incision in the abdomen?

□	Yes

□	No

# 20. How many of these hernias do you encounter per year in your practice?

□	0 – 5 hernias

□	5 – 10 hernias

□	> 10 hernias

# 21. In your experience, are hernias following a lower transverse incision in the abdomen most commonly:

□	Partial (intraparietal – herniation between layers of the abdominal wall)

□	Total (true incisional – herniation beyond all layers of the abdominal wall)

□	Mixed

# 22. In your experience, are hernias from a lower transverse incision often detected on CT scans?

□	Commonly reported, with findings as visible on scan

□	Commonly reported, with findings different from those visible on scan

□	Commonly not reported

# 23. Have you ever incidentally found a lower transverse incision hernia during a laparoscopic procedure?

□	Yes

□	No

# 24. Many lower transverse incision hernias are intraparietal and only mildly symptomatic. In your opinion are these hernias overlooked and/or underreported?

□	Yes

□	No

**Appendix 2: Demographics and Experience Compared by Respondent Subspecialty**

|  |  | General Surgery (n=158) | Urology (n=12) | Obstetrics & Gynaecology (n=61) | p value |
| --- | --- | --- | --- | --- | --- |
| Age (years) | Median (IQR) | 48.0  (41.0 to 56.0) | 40.5  (38.2 to 43.8) | 44.0  (37.5 to 53.3) | 0.022 |
| Gender | Male | 126 (79.7) | 12 (100.0) | 21 (34.4) |  |
|  | Female | 32 (20.3) | 0 (0.0) | 40 (65.6) | <0.001 |
| Country Income | HIC | 73 (46.2) | 3 (25.0) | 40 (65.6) | 0.033 |
|  | UMIC | 65 (41.) | 7 (58.3) | 18 (29.5) |  |
|  | LMIC | 20 (12.7) | 2 (16.7) | 3 (4.9) |  |
| Consultant Experience (years) | Median (IQR) | 17.0  (7.2 to 24.0) | 6.5  (1.8 to 18.5) | 9.0  (2.0 to 18.0) | 0.001 |
| LATI Case Frequency  (cases / year) | 0 – 10 | 103 (65.2) | 6 (50.0) | 6 (9.8) | <0.001 |
|  | 10 – 20 | 23 (14.6) | 4 (33.3.) | 5 (8.2) |  |
|  | 20 – 50 | 21 (13.3.) | 2 (16.7) | 13 (21.3) |  |
|  | >50 | 11 (7.0) | 0 (0.0) | 37 (60.7) |  |

Data are displayed as number (%) unless stated otherwise. HIC: High Income Country, UMIC: Upper-Middle Income Country, LMIC: Lower-Middle Income Country. LATI: Lower Abdominal Transverse Incision. Experience reported as years in independent consultant practice.

**Appendix 3: Demographics and Experience Compared by Country Income Status**

|  |  | High Income  (n=116) | Upper-Middle Income (n=90) | Lower-Middle Income  (n=25) | p  value |
| --- | --- | --- | --- | --- | --- |
| Age (years) | Median (IQR) | 45.0 (39.0 to 53.0) | 47.5 (42.2 to 57.8) | 45.0 (36.0 to 55.0) | 0.211 |
| Gender | Male | 70 (60.3) | 72 (80.0) | 17 (68.0) | 0.010 |
|  | Female | 46 (39.7) | 18 (20.0) | 8 (32.0) |  |
| Specialty | Colorectal | 12 (10.3) | 5 (5.6) | 2 (8.0) | 0.012 |
|  | Upper GI | 6 (5.2) | 18 (20.0) | 3 (12.0) |  |
|  | Hernia | 46 (39.7) | 30 (33.3) | 10 (40.0) |  |
|  | Emergency | 2 (1.7) | 6 (6.7) | 2 (8.0) |  |
|  | Other General | 7 (6.0) | 6 (6.7) | 3 (12.0) |  |
|  | Urology | 3 (2.6) | 7 (7.8) | 2 (8.0) |  |
|  | O&G | 40 (34.5) | 18 (20.0) | 3 (12.0) |  |
| Consultant Experience (years) | Median (IQR) | 8.5 (3.0 to 19.0) | 20.0 (11.0 to 30.0) | 15.0 (7.0 to 25.0) | <0.001 |
| LATI Case Frequency  (cases / year) | 0 – 10 | 53 (45.7) | 52 (57.8) | 10 (40.0) | <0.001 |
|  | 10 – 20 | 10 (8.6) | 14 (15.6) | 8 (32.0) |  |
|  | 20 – 50 | 17 (14.7) | 13 (14.4) | 6 (24.0) |  |
|  | >50 | 36 (31.0) | 11 (12.2) | 1 (4.0) |  |

Data are displayed as number (%) unless stated otherwise. GI: Gastrointestinal. O&G: Obstetrics and Gynaecology. LATI: Lower Abdominal Transverse Incision. Experience reported as years in independent consultant practice*.*

**Appendix 4: LATI Entry Technique Compared by Subspecialty**

|  |  | **General Surgery (n=158)** | **Urology  (n=12)** | **Obstetrics & Gynaecology (n=61)** | ***p*  value** |
| --- | --- | --- | --- | --- | --- |
| **Open Fascia** | Blunt | 3 (1.9) | 0 (0.0) | 10 (16.4) | <0.001 |
|  | Sharp | 24 (15.2) | 1 (8.3) | 14 (23.0) |  |
|  | Diathermy | 91 (57.6) | 8 (66.7) | 8 (13.1) |  |
|  | Combination | 40 (25.3) | 3 (25.0) | 29 (47.5) |  |
| **Open Rectus** | Cut partly | 9 (5.7) | 0 (0.0) | 1 (1.6) | 0.023 |
|  | Cut entirely | 1 (0.6) | 0 (0.0) | 3 (4.9) |  |
|  | Separate detach | 82 (51.9) | 10 (83.3) | 41 (67.2) |  |
|  | Separate without | 66 (41.8) | 2 (16.7) | 16 (26.2) |  |
| **Open Peritoneum** | Blunt | 11 (7.0) | 2 (16.7) | 38 (62.3) | <0.001 |
|  | Sharp | 71 (44.9) | 4 (33.3) | 12 (19.7) |  |
|  | Diathermy | 46 (29.1) | 2 (16.7) | 2 (3.3) |  |
|  | Combination | 30 (19.0) | 4 (33.3) | 9 (14.8) |  |
| **Peritoneum Direction** | Transverse | 25 (15.8) | 2 (16.7) | 33 (54.1) | <0.001 |
|  | Longitudinal | 133 (84.2) | 10 (83.3) | 28 (45.9) |  |

Data are displayed as number (%) unless stated otherwise.

**Appendix 5: LATI Entry Technique Compared by Country Income Status**

|  |  | **High Income  (n=116)** | **Upper-Middle Income (n=90)** | **Lower-Middle Income (n=25)** | ***p***  **value** |
| --- | --- | --- | --- | --- | --- |
| **Open Fascia** | Blunt | 8 (6.9) | 5 (5.6) | 0 (0.0) | 0.561 |
|  | Sharp | 16 (13.8) | 18 (20.0) | 5 (20.0) |  |
|  | Diathermy | 58 (50.0) | 36 (40.0) | 13 (52.0) |  |
|  | Combination | 34 (29.3) | 31 (34.4) | 7 (28.0) |  |
| **Open Rectus** | Cut partly | 4 (3.4) | 6 (6.7) | 0 (0.0) | 0.383 |
|  | Cut entirely | 3 (2.6) | 1 (1.1) | 0 (0.0) |  |
|  | Separate detach | 61 (52.6) | 56 (62.2) | 16 (64.0) |  |
|  | Separate without | 48 (41.4) | 27 (30.0) | 9 (36.0) |  |
| **Open Peritoneum** | Blunt | 36 (31.0) | 14 (15.6) | 1 (4.0) | 0.002 |
|  | Sharp | 36 (31.0) | 35 (38.9) | 16 (64.0) |  |
|  | Diathermy | 28 (24.1) | 18 (20.0) | 4 (16.0) |  |
|  | Combination | 16 (13.8) | 23 (25.6) | 4 (16.0) |  |
| **Peritoneum Direction** | Transverse | 39 (33.6) | 16 (17.8) | 5 (20.0) | 0.028 |
|  | Longitudinal | 77 (66.4) | 74 (82.2) | 20 (80.0) |  |

Data are displayed as number (%) unless stated otherwise.

**Appendix 6: LATI Closure Technique Compared by Subspecialty**

|  |  | **General surgery (n=158)** | **Urology  (n=12)** | **Obstetrics & Gynaecology (n=61)** | ***p*  value** |
| --- | --- | --- | --- | --- | --- |
| **Peritoneum Closure** | Yes | 123 (77.8) | 6 (50.0) | 14 (23.0) | <0.001 |
|  | No | 35 (22.2) | 6 (50) | 47 (77.0) |  |
| **Suture Peritoneum** | Fast absorbable | 58 (36.7) | 4 (33.3) | 15 (24.6) | 0.283 |
|  | Slowly absorbable | 68 (43.0) | 3 (25.0) | 6 (9.8) |  |
|  | Non-absorbable | 1 (0.6) | 0 (0.0) | 0 (0.0) |  |
|  | (Missing) | 31 (19.6) | 5 (41.7) | 40 (65.6) |  |
| **Close Rectus** | Yes | 89 (56.3) | 10 (83.3) | 22 (36.1) | 0.002 |
|  | No | 69 (43.7) | 2 (16.7) | 39 (63.9) |  |
| **Suture Rectus** | Fast absorbable | 40 (25.3) | 5 (41.7) | 15 (24.6) | 0.458 |
|  | Slowly absorbable | 55 (34.8) | 5 (41.7) | 9 (14.8) |  |
|  | Non-absorbable | 1 (0.6) | 0 (0.0) | 0 (0.0) |  |
|  | (Missing) | 62 (39.2) | 2 (16.7) | 37 (60.7) |  |
| **Close Fascia** | Continuous | 154 (97.5) | 11 (91.7) | 57 (93.4) | 0.277 |
|  | Interrupted | 4 (2.5) | 1 (8.3) | 4 (6.6) |  |
| **Fascia Method** | Small bites | 138 (87.3) | 7 (58.3) | 26 (42.6) | <0.001 |
|  | Large bites | 16 (10.1) | 5 (41.7) | 32 (52.5) |  |
|  | (Missing) | 4 (2.5) | 0 (0.0) | 3 (4.9) |  |
| **Fascia Suture** | Fast absorbable | 2 (1.3) | 0 (0.0) | 11 (18.0) | <0.001 |
|  | Slowly absorbable | 130 (82.3) | 11 (91.7) | 49 (80.3) |  |
|  | Non-absorbable | 26 (16.5) | 1 (8.3) | 1 (1.6) |  |

Data are displayed as number (%) unless stated otherwise.

**Appendix 7: LATI Closure Technique Compared by Country Income Status**

|  |  | **High Income**  **(n=116)** | **Upper-Middle Income (n=90)** | **Lower-Middle Income (n=25)** | ***p*  value** |
| --- | --- | --- | --- | --- | --- |
| **Peritoneum Close** | Yes | 69 (59.5) | 54 (60.0) | 20 (80.0) | 0.142 |
|  | No | 47 (40.5) | 36 (40.0) | 5 (20.0) |  |
| **Suture Peritoneum** | Fast absorbable | 35 (30.2) | 35 (38.9) | 7 (28.0) | 0.358 |
|  | Slowly absorbable | 38 (32.8) | 26 (28.9) | 13 (52.0) |  |
|  | Non-absorbable | 1 (0.9) | 0 (0.0) | 0 (0.0) |  |
|  | (Missing) | 42 (36.2) | 29 (32.2) | 5 (20.00 |  |
| **Close Rectus** | Yes | 39 (33.6) | 68 (75.6) | 14 (56.0) | <0.001 |
|  | No | 77 (66.4) | 22 (24.4) | 11 (44.0) |  |
| **Suture Rectus** | Fast absorbable | 20 (17.2) | 31 (34.4) | 9 (36.0) | 0.709 |
|  | Slowly absorbable | 23 (19.8) | 40 (44.4) | 6 (24.0) |  |
|  | Non-absorbable | 0 (0.0) | 1 (1.1) | 0 (0.0) |  |
|  | (Missing) | 73 (62.9) | 18 (20.0) | 10 (40.0) |  |
| **Close Fascia** | Continuous | 114 (98.3) | 84 (93.3) | 24 (96.0) | 0.191 |
|  | Interrupted | 2 (1.7) | 6 (6.7) | 1 (4.0) |  |
| **Fascia Method** | Small bites | 85 (73.3) | 69 (76.7) | 17 (68.0) | 0.732 |
|  | Large bites | 27 (23.3) | 19 (21.1) | 7 (28.0) |  |
|  | (Missing) | 4 (3.4) | 2 (2.2) | 1 (4.0) |  |
| **Fascia Suture** | Fast absorbable | 10 (8.6) | 2 (2.2) | 1 (4.0) | <0.001 |
|  | Slowly absorbable | 104 (89.7) | 65 (72.2) | 21 (84.0) |  |
|  | Non-absorbable | 2 (1.7) | 23 (25.6) | 3 (12.0) |  |

Data are displayed as number (%) unless stated otherwise.

**Appendix 8: LATI Hernia Experience Compared by Country Income Status**

|  |  | **High Income**  **(n=116)** | **Upper-Middle Income (n=90)** | **Lower-Middle Income (n=25)** | ***p***  **value** |
| --- | --- | --- | --- | --- | --- |
| **LATI Hernia Seen** | Yes | 86 (74.1) | 66 (73.3) | 14 (56.0) | 0.173 |
|  | No | 30 (25.9) | 24 (26.7) | 11 (44.0) |  |
| **Number of LATI** | 0 - 5 hernias | 87 (75.0) | 69 (76.7) | 18 (72.0) | 0.711 |
| **Hernias seen** | 5 - 10 hernias | 16 (13.8) | 13 (14.4) | 3 (12.0) |  |
| **(per year)** | >10 hernias | 8 (6.9) | 7 (7.8) | 4 (16.0) |  |
|  | (Missing) | 5 (4.3) | 1 (1.1) | 0 (0.0) |  |
| **Hernia Type Seen** | Total | 15 (12.9) | 16 (17.8) | 4 (16.0) | 0.410 |
|  | Partial | 45 (38.8) | 46 (51.1) | 10 (40.0) |  |
|  | Mixed | 46 (39.7) | 27 (30.0) | 11 (44.0) |  |
|  | (Missing) | 10 (8.6) | 1 (1.1) | 0 (0.0) |  |
| **LATI Hernia Found (CT Imaging)** | Commonly not reported | 58 (50.0) | 40 (44.4) | 7 (28.0) | 0.104 |
|  | Commonly reported (report not as visualised) | 17 (14.7) | 12 (13.3) | 6 (24.0) |  |
|  | Commonly reported (report as visualised) | 30 (25.9) | 37 (41.1) | 11 (44.0) |  |
|  | (Missing) | 11 (9.5) | 1 (1.1) | 1 (4.0) |  |
| **LATI Hernia Found** | Yes | 63 (54.3) | 46 (51.1) | 11 (44.0) | 0.638 |
| **(Laparoscopy)** | No | 50 (43.1) | 43 (47.8) | 13 (52.0) |  |
|  | (Missing) | 3 (2.6) | 1 (1.1) | 1 (4.0) |  |
| **Are LATI Hernias** | Yes | 93 (80.2) | 80 (88.9) | 21 (84.0) | 0.678 |
| **Underreported** | No | 14 (12.1) | 9 (10.0) | 4 (16.0) |  |
|  | (Missing) | 9 (7.8) | 1 (1.1) | 0 (0.0) |  |

Data are displayed as number (%) unless stated otherwise. CT: Computed Tomography. LATI: Lower Abdominal Transverse Incision.
